# Supplementary material for: Causal Relationship between Aging and Anorexia Nervosa: A White-Matter-Microstructure-Mediated Mendelian Randomization Analysis
Source: Biomedicines. 2024 Aug 16;12(8):1874. doi: 10.3390/biomedicines12081874 (PMC11351342; doi:10.3390/biomedicines12081874)
Supplement: Supplementary file 1 [file biomedicines-12-01874-s001.zip › Supplementary Figure/Figure S8/Leaveoneout plot of MR analysis for TL to ubm-b-1470.pdf]

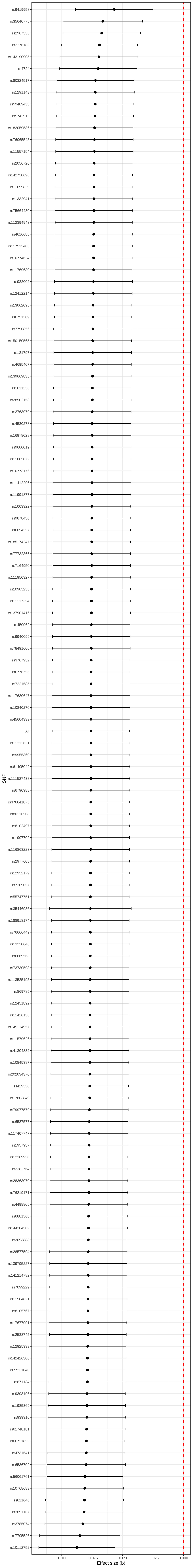

rs9419958
rs35640778
rs2967355
rs2276182
rs143190905
rs4724
rs80324517
rs1291143
rs59409453
rs5742915
rs182059586
rs76065543
rs11557154
rs2056726
rs142730696
rs11699829
rs1332941
rs75664430
rs112394943
rs4616688
rs117512405
rs10774624
rs11769630
rs932002
rs12412214
rs13062095
rs6751209
rs7790856
rs150150565
rs131797
rs4695407
rs139669835
rs1611236
rs28502153
rs2763979
rs4530278
rs16978028
rs9600019
rs11085072
rs10773176
rs11412296
rs11991877
rs1003322
rs9878436
rs6054257
rs185174247
rs77732866
rs7164950
rs111950327
rs10905255
rs11117354
rs137901416
rs450962
rs9940099
rs78491606
rs3767952
rs6776756
rs7221585
rs117630647
rs10840270
rs45604339
All
rs11212631
rs9955360
rs61405042
rs111527438
rs6790988
rs376641875
rs80116508
rs8102497
rs1907702
rs116863223
rs2977608
rs12932179
rs7209057
rs55747751
rs35446936
rs188918174
rs76666449
rs13230646
rs6669563
rs73730598
rs113525195
rs869785
rs12451892
rs11426156
rs145114957
rs11579626
rs41304832
rs10845387
rs202034370
rs429358
rs17803849
rs79977579
rs6587577
rs117407747
rs1957937
rs12369950
rs2282764
rs28363070
rs76219171
rs4498805
rs6881568
rs144204502
rs3093888
rs28577594
rs139795227
rs141214782
rs7099229
rs11584821
rs8105767
rs17677991
rs2538745
rs12925933
rs142426306
rs77231040
rs871134
rs9398196
rs1985369
rs939916
rs61748181
rs66731853
rs4731541
rs6536702
rs56061761
rs10768683
rs611646
rs3891167
rs3785074
rs7705526
rs10112752
SNP
−0.100
−0.075
−0.050
−0.025
0.000
Effect size (b)
